# The binding landscape of a partially-selective isopeptidase inhibitor with potent pro-death activity, based on the bis(arylidene)cyclohexanone scaffold

**DOI:** 10.1038/s41419-017-0259-1

**Published:** 2018-02-07

**Authors:** Sonia Ciotti, Riccardo Sgarra, Andrea Sgorbissa, Carlotta Penzo, Andrea Tomasella, Federico Casarsa, Fabio Benedetti, Federico Berti, Guidalberto Manfioletti, Claudio Brancolini

**Affiliations:** 1 Department of Medicine, Università degli Studi di Udine, P.le Kolbe 4, 33100 Udine, Italy; 2 Department of Life Sciences, Università degli Studi di Trieste, Via L. Giorgieri 5, 34127 Trieste, Italy; 3 Dipartimento di Scienze Chimiche e Farmaceutiche, Universita degli Studi di Trieste, Via Giorgieri 1, 34127 Trieste, Italy

## Abstract

Diaryldienone derivatives with accessible β-carbons show strong anti-neoplastic properties, related to their ability to make covalent adducts with free thiols by Michael addition, and low toxicity in vivo. Accumulation of poly-ubiquitylated proteins, activation of the unfolded protein response (UPR) and induction of cell death are universal hallmarks of their activities. These compounds have been characterized as inhibitors of isopeptidases, a family of cysteine-proteases, which de-conjugate ubiquitin and ubiquitin-like proteins from their targets. However, it is unclear whether they can also react with additional proteins. In this work, we utilized the biotin-conjugated diaryldienone-derivative named 2c, as a bait to purify novel cellular targets of these small molecules. Proteomic analyses have unveiled that, in addition to isopeptidases, these inhibitors can form stable covalent adducts with different intracellular proteins, thus potentially impacting on multiple functions of the cells, from cytoskeletal organization to metabolism. These widespread activities can explain the ability of diaryldienone derivatives to efficiently trigger different cell death pathways.

## Introduction

During the past decades the identification of new small molecule therapeutics has provided some improvements for clinical treatments in patients with various tumors. However, for certain cancers and particularly solid tumors, exhibiting extreme drug resistance, the demand of new agents is still urgent. A class of small organic molecules, which derive from diaryldienone and contain cross-conjugated α,β-unsaturated ketones with accessible β-carbons, have evidenced anti-neoplastic properties and low toxicity in different preclinical studies in vivo^1–6^. The β carbon atoms of α,β-unsaturated ketones are available to alkylate various cellular nucleophiles^6–9^. These small molecules have been proposed as non-selective inhibitors of isopeptidases, a family of enzymes that are involved in the de-conjugation of ubiquitin and ubiquitin-like proteins from different targets^1,10^. Indeed, cells treated with these molecules accumulate poly-ubiquitylated proteins in the presence of unperturbed proteasomal catalytic activity^1,2,5,10,11^. Since several isopeptidases are cysteine-proteases, they are prone to react with the α,β-unsaturated ketones, thus leading to enzyme inactivation. Importantly, these compounds cannot efficiently inhibit the activity of other cysteine proteases, such as caspases or cathepsins, thus indicating a certain degree of specificity^9^.

In vitro they can inhibit the activity of recombinant isopeptidases^8,12^ and the presence of different groups, in addition to the pharmacophore, can modulate the promiscuity of these compounds^9,13^. Hence, we refer to them as partially-selective isopeptidase inhibitors (P-SIIs).

In cells treated with these P-SIIs, accumulation of poly-ubiquitylated proteins is evident, in partial analogy to bortezomib treatment^2,14^. Bortezomid/Carfilzomib are inhibitors of the proteasomal catalytic chamber approved for the use in clinic^9^. For this reason P-SIIs are usually considered as alternative proteasome inhibitors. However, when the cellular responses to the two inhibitors are compared by gene expression profile studies, the signatures are not entirely superimposable. For example, 2c, a P-SII previously identified and characterized^8^, exhibits broader effects in terms of activation of adaptive responses. The response to oxidative stress and protein misfolding are more pronounced in 2c compared to bortezomib treated cells^14^.

The identification of the cellular targets of compounds with therapeutic potential is an important step to improve their activities and to understand potential side effects. Chemical proteomics is an innovative approach to unmask the cellular targets of small molecule therapeutics. In chemical proteomics, affinity chromatography is combined with proteomic techniques such as mass spectrometry for the unbiased identification of protein targets^15^. To identify the cellular target of P-SIIs and to clarify their selectivity, we isolated cellular proteins bound by 2c and analysed them by mass spectrometry. Our approach employed a 2c-biotin conjugate as a probe to identify in vivo the cellular targets of P-SIIs.

## Results

### Generation of the 2c-biotinylated probe

**Fig. 1:**
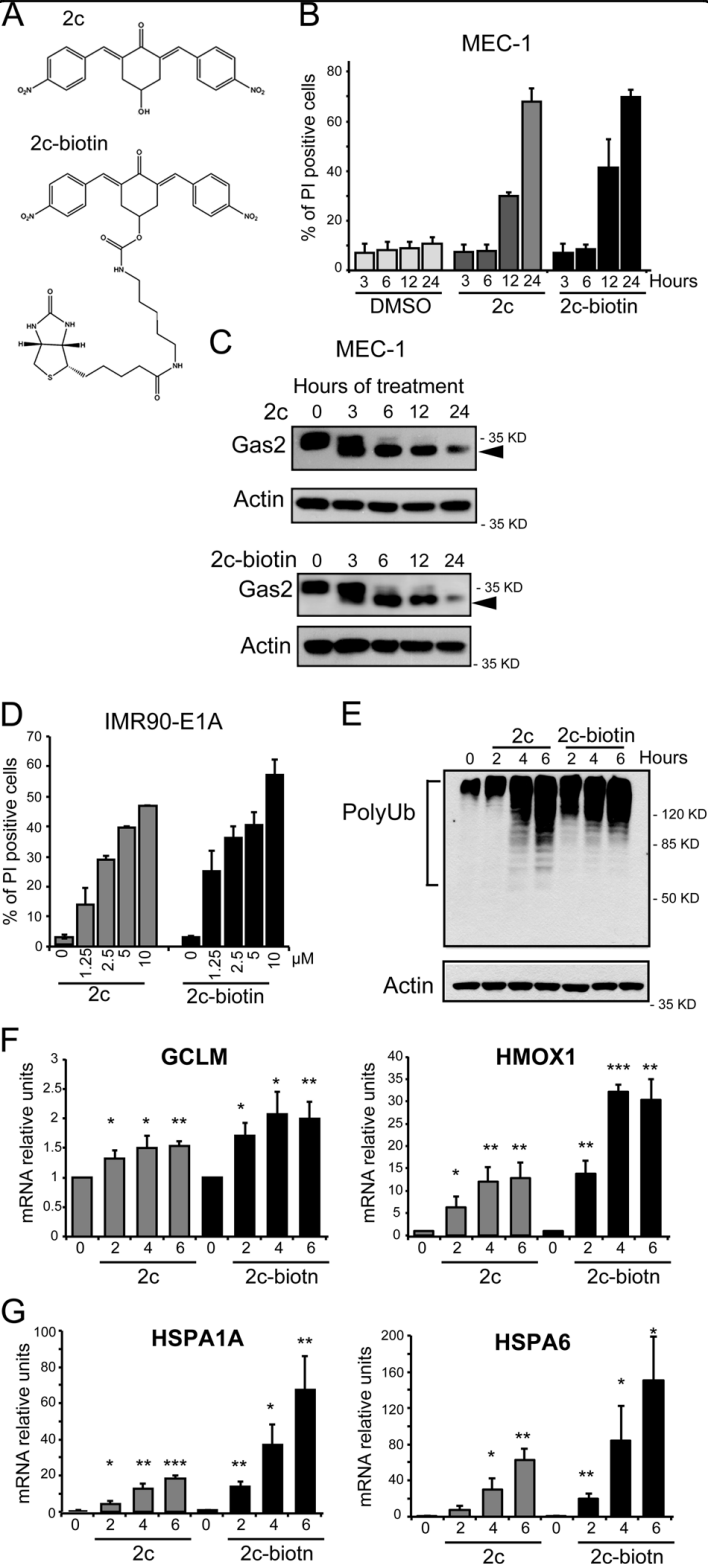
Generation and characterization of biotin-conjugated 2c. **a** Structure of 2c and of the 2c-biotin conjugate. **b** MEC-1 cells were treated for the indicated times with 2 µM 2c, 2c-biotin, or DMSO alone as control. After 24 h cells were stained wit PI fixed and processed for cytofluorimetric analysis. Mean ± SD; *n* = 3. **c** Cellular lysates of MEC-1 cells treated with 2 µM 2c or of 2c-biotin for the indicated times were subjected to immunoblotting using the anti-Gas2 antibody. Actin immuno-detection was used as loading control. Arrowhead points to the caspase-cleaved product. **d** IMR90-E1A cells were treated with the indicated concentrations of 2c, 2c-biotin, or DMSO as control. After 24 h cells were stained wit PI fixed and processed for cytofluorimetric analysis. Mean ± SD; *n* = 3. **e** Cellular lysates of IMR90-E1A cells treated with 10 µM 2c or 2c-biotin for the indicated times were subjected to immunoblotting using the anti-ubiquitin antibody. Actin was used as loading control. **f** qRT-PCR analysis of mRNA expression levels for GLCM and HMOX1 in cells treated for the indicated hours with 10 µM 2c or 2c-biotin. Mean ± SD; *n* = 3. **g** qRT-PCR analysis of mRNA expression levels of HSPA1A and HSPA6 in cells treated for the indicated hours with 10 µM 2c or 2c-biotin. Mean ± SD; *n* = 3

In order to better characterize the mechanisms through which, diaryldienone-derivatives P-SIIs trigger cell death and to define their cellular targets, we generated a biotinylated probe of 2c (Fig. 1). 2c is a P-SII that we have recently synthetized and optimized for in vivo delivery^8^. We initially compared the ability of 2c-biotin, with respect to the original compound, of triggering cell death. MEC-1 chronic lymphocytic leukemia cells were treated for different times with the two compounds and the appearance of cell death was evaluated by cytofluorimetric analysis. Cell death was similarly induced by 2c and its biotinylated version (Fig. 1b). Next, we verified that the death response occurred through the activation of apoptosis. Caspase-dependent processing of GAS2 was detected as early as after 3 h of treatment and was also comparable for the two compounds (Fig. 1c)^16^. Finally, we also compared the pro-death activities of 2c and of 2c-biotin in a different cell line. IMR90-E1A cells evidence a similar dose-dependent response, when challenged with the two compounds (Fig. 1d). In summary, biotinylation of 2c does not perturb its ability to trigger cell death.

### Biotinylation does not alter the biological properties of 2c

Cell death induced by 2c and similar compounds is characterized by: (i) the accumulation of poly-ubiquitylated proteins, (ii) the induction of oxidative stress response and (iii) of the unfolded protein response (UPR)^1,8,14,17^. To verify that the presence of the biotin tag does not influence the activation of these cellular responses, we initially investigated the accumulation of polyubiquitylated proteins. Figure 1e illustrates that both 2c and 2c-biotin elicit the accumulation of poly-ubiquitylated proteins. Data suggest that the original compound is slightly more efficient in blocking proteasomal deubiquitylase activities. To evaluate the up-regulations of the oxidative stress signaling, qRT-PCR analysis was performed to measure the relative mRNA levels of heme oxygenase HMOX1 and of GCLM, the regulative subunit of the first rate-limiting enzyme in glutathione biosynthesis^14^. The expression of both genes was upregulated in response to both 2c and 2c-biotin. In this instance, the mRNA levels of both genes were higher in cells treated with the biotinylated 2c (Fig. 1f). Finally, we also investigated the upregulation of the UPR (Unfolded Protein Response). We previously observed that treatment with 2c triggers the upregulation of several chaperons, including HSPA1A and HSPA6^14^. Figure 1g illustrates that the mRNA levels of HSPA1A and HSPA6 were both increased in IMR90-E1A cells treated with 2c or its biotinylated analog. However, this upregulation was stronger in the case of 2c-biotin. In summary, our studies indicate that 2c and 2c-biotin elicit similar adaptive cellular responses, even though some specificity can be appreciated.

### Nuclear accumulation of 2c-biotin

**Fig. 2:**
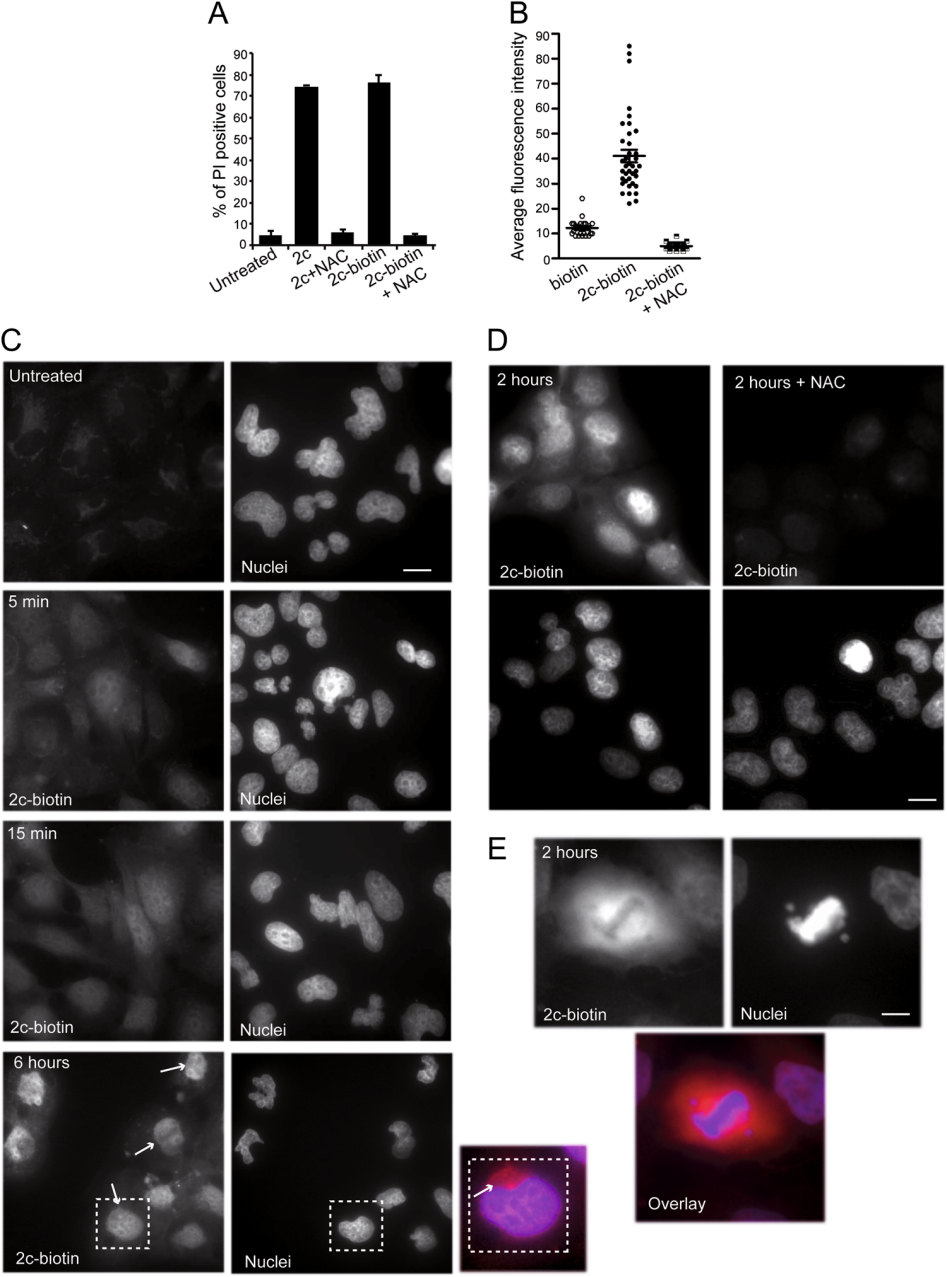
Dynamics of 2c-biotin uptake and accumulation in cells. **a** IMR90-E1A cells were treated for 24 h as indicated. Cell death was scored as PI positivity by cytofluorimetric analysis. 2c and 2c-biotin were used at 10 µM. NAC was 2 mM. Mean ± SD; *n* = 3. **b** IMR90-E1A cells grown on coverslips were treated for 6 h as indicated. Cells were fixed and analysed with confocal microscope using the same parameters of acquisition. 2c-biotin was revealed using streptavidin-TRITC. Quantitative analysis of the fluorescence intensity was performed using the Metamorph software. **c** IMR90-E1A cells were treated with 2c-biotin [2 µM] for the indicated times. After fixation cells were incubated with streptavidin-TRITC. Nuclei were visualized using Hoechst 33,342. Arrows point to cytosolic accumulation of 2c-biotin. The dotted square indicates the enlarged area. Images were obtained with an epifluorescence microscopy. Bar 30 µm. **d** IMR90-E1A cells were treated with 2c-biotin [2 µM] for 2 h. When used in pre-treatment, NAC was 2 mM. After fixation cells were incubated with streptavidin-TRITC. Nuclei were visualized using Hoechst 33,342. Images were obtained with an epifluorescence microscopy. Bar 30 µm. **e** IMR90-E1A cells were treated with 2c-biotin [2 µM] for 2 h. After fixation cells were incubated with streptavidin-TRITC. Nuclei were visualized using Hoechst 33,342. Images were obtained with an epifluorescence microscopy. A mitotic cell is shown. Bar 50 µm. Images are shown in pseudocolors

2c and similar compounds, containing electrophilic α,β-unsaturated carbonyl units as Michael acceptors, show very high and selective affinity for thiol residues, in particular for cysteines. Impairment of the α, β-unsaturated carbonyl reactivity in these compounds results in a strong decrease of their pro-death potency^6,8,18^. Accordingly, when 2c or 2c-biotin were pre-incubated with N-acetyl cysteine (NAC) their pro-death activity was suppressed (Fig. 2a). Hence, we used NAC as a tool to evaluate the specificity of 2c-biotin activities in vivo.

Initially, cells were treated with biotin alone, 2c-biotin, or 2c-biotin pre-incubated with NAC. After 6 h cells were fixed and processed for fluorescence analysis using streptavidin-tetramethylrhodamine (TRITC). Confocal images were acquired and then analysed for fluorescence intensity. Fluorescence was dramatically augmented when cells were treated with 2c-biotin and this fluorescence was suppressed when the compound was pre-incubated with NAC (Fig. 2b).

Next we performed a time-course analysis to visualize variations of the subcellular localization of 2c-biotin with time. 2c-biotin positivity was detectable as soon as 5 min after treatment and fluorescence was evident in the nucleus and also in the cytoplasm (Fig. 2c). After 10 min the intensity of fluorescence was further increased. At later time-points (6 h) some signs of cellular stress, such as reduced cell spreading, were evident. At this time nuclear localization of 2c-biotin was even stronger, whereas in the cytoplasm it accumulates in a perinuclear area (see arrows and the higher magnification).

Pre-treatment with NAC completely abolishes the fluorescent signals. This behavior suggests that covalent binding of 2c to cellular thiols is necessary for its intracellular accumulation or that the NAC-2c complex is not transported through the plasma membrane. thus confirming that covalent binding of 2c to cellular thiols is necessary for its intracellular accumulation (Fig. 2d). Finally, analysis of mitotic cells suggests that 2c does not interact with chromatin, being chromosomes in metaphase negative for 2c-biotin signals (Fig. 2e).

### Intracellular targets of the PS-II 2c

In order to identify PS-IIs intracellular targets, IMR90-E1A cells were treated with 2c-biotin and, after lysis, cellular extracts were incubated with streptavidin beads. NAC was added to verify that binding of the targets was due to the Michael acceptor activity of the probe. To evaluate the binding to isopeptidases, we performed immunoblot analysis on proteins purified after 2c-biotin mediated pull-down. For enrichment comparison, each pull-down was incubated in parallel with antibodies against unrelated proteins.

**Fig. 3:**
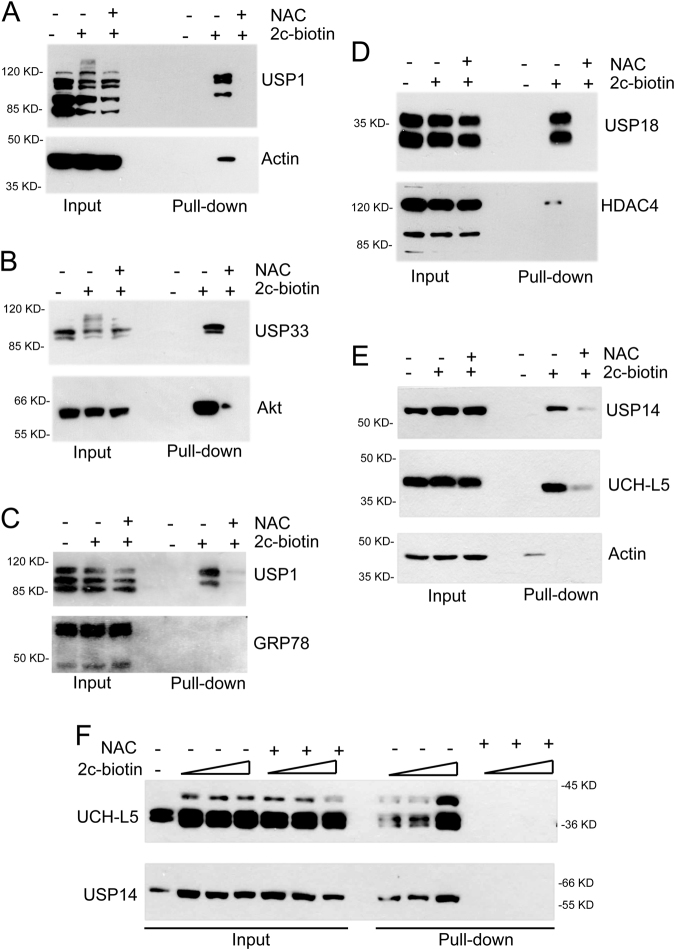
Identification of target isopeptidases by biotin pull-down experiments. **a/b/c/d/e** IMR90-E1A cells were treated for 2 h with 10 µM 2c-biotin or left untreated. 2c-botin was pre-incubated with 5 mM of NAC, as a negative control. Cellular proteins were purified by 2c-biotin pull-down using streptavidin-agarose beads. Protein complexes were separated by SDS/PAGE and immunoblotted using the indicated antibodies. Inputs are included for enrichment comparison. In the case of USP18 cells were pre-treated for 24 h with interferon-α. **f** IMR90-E1A cells were treated for 2 h with the indicated concentrations of 2c-biotin or left untreated. As a control, 2c-biotin was pre-incubated with 5 mM NAC. Cellular proteins were purified by 2c-biotin pull-down. Protein complexes were separated by SDS/PAGE and immunoblotted using the anti-USP14 or the anti-UCH-L5 antibodies. Inputs are included for enrichment comparison

We initially evaluated the enrichments of USP1 and of actin, used as negative control, with respect to their inputs. USP1 signal was highly enriched in a NAC-dependent manner after 2c-biotin purification (Fig. 3a). In the case of actin, only a weak signal was observed in the 2c pull-down. Next, we analysed the binding of 2c-biotin to USP33 and to the serine-threonine kinase AKT, for comparison (Fig. 3b). USP33, similarly to USP1, was enriched after 2c-biotin pull-down in a NAC dependent manner. Surprisingly, also AKT, after incubation with 2c-biotin was enriched. Hence, we repeated the experiment with USP1 using a different control, the chaperon GRP78. USP1 was again enriched, whereas GRP78 was almost undetectable in the 2c-biotin pull-down (Fig. 3c). Since previous studies showed that 2c inhibits the deISGlase activity of USP18, we also evaluated the 2c pull-down for USP18^8^. Here as control we used HDAC4. Binding to USP18 was evident whereas binding of HDAC4 was very weak (Fig. 3d).

Finally, we compared the binding of 2c to UCH-L5 and USP14, the two cysteine-deubiquitylases of the proteasome. Their inhibition should represent the key contribution to the accumulation of poly-ubiquitylated proteins^9,13^. The pull-down of both DUBs was enriched compared to Actin (Fig. 3e). Previous studies, using different assays, indicated that compounds similar to 2c exhibit higher inhibition of USP14 activity compared to UCH-L5. Hence, we compared in more detail the binding of 2c-biotin to the two DUBs using different concentrations of the inhibitor. Figure 3f confirms that in vivo 2c-biotin binds with comparable intensity USP14 and UCH-L5.

In summary, we demonstrated that the compound 2c shows a certain affinity for isopeptidases. The spectrum of enzymes recognized by 2c seems to be quite wide, thus confirming its partial selectivity. However, USP1/USP33 showed a higher affinity when compared to USP18/UCH-L5/USP14. As expected, unrelated control proteins such as Actin, HDAC4 and GRP78 showed a weak reaction with 2c-biotin. Surprisingly, AKT can be covalently modified by 2c-biotin.

### Proteomic profiling of 2c adducts in transformed cells

The above study suggests that, in addition to isopeptidases, 2c might target additional proteins such as AKT. To characterize the binding landscape of 2c, we applied a chemical proteomic strategy. IMR90-E1A cells were treated with 10 µM 2c-biotin for 2 h and lysed. Biotinylated proteins were then isolated using avidin column chromatography. As a control, the same experiment was performed in the presence of NAC. After SDS-PAGE separation and blue coomassie staining, lanes were divided in five parts, excised and trypsin digested. The resulting peptides were analyzed by liquid chromatography coupled to tandem mass spectrometry (LC–MS/MS) and 25 proteins were successfully identified.

**Fig. 4:**
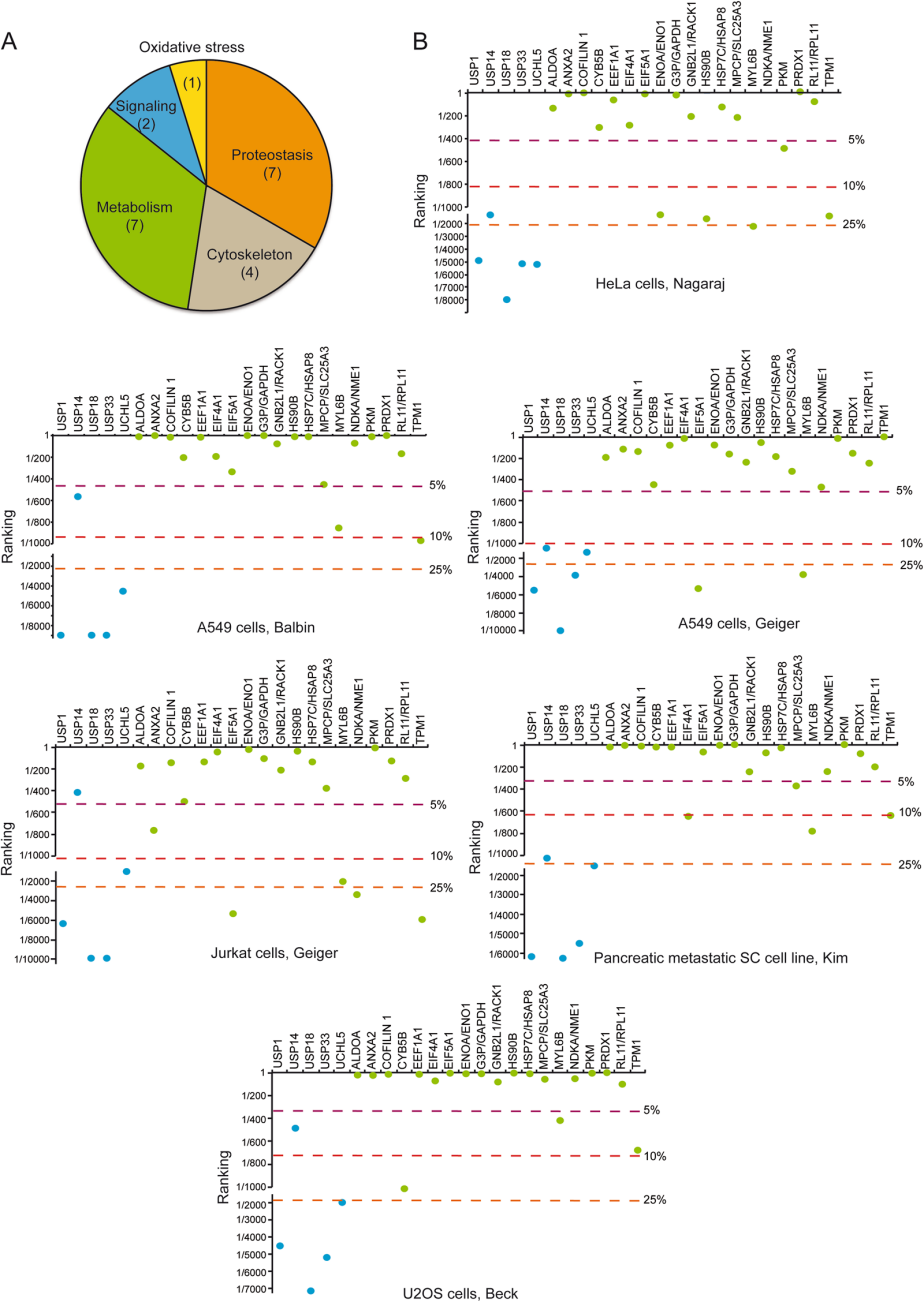
Candidate 2c-biotin targets identified by mass-spectrometry. **a** Identified proteins can be grouped into categories: metabolism, proteostasis, cytoskeleton, oxidative stress, and signaling. Numbers of proteins of each category are indicated. **b** Relative abundance of the isopeptidases evaluated for 2c-biotin binding by immunoblots (blue dots) in comparison with proteins identified by mass-spectrometry (green dots). Data are presented as ranking. Data were obtained from http://pax-db.org. The selected cell lines and the original datasets are indicated

Table 1 summarizes the results of such analysis. The identified proteins fall into five different functional classes including; metabolism (33%), proteostasis (33%), cytoskeleton (19%), signaling (10%) and oxidative stress (5%) (Fig. 4a). As expected some proteins were detected after the NAC treatment as well. They could represent contaminants such as Tubulin and Actin. Hence, we excluded them from the analysis. Pyruvate kinase and Eukaryotic initiation factor 4A-1 were the two proteins detected with the highest scores. To clarify whether the 2c-biotin modified proteins, identified by mass-spectrometry, reflect their relative abundance, we interrogated the protein abundance database PaxDb (http://pax-db.org.)^19^. We also compared the abundance of the isopeptidases USP1, USP14, USP18 USP33 and UCH-L5. Different cell lines and different datasets were scrutinized^20–24^. Figure 4b shows the result of this analysis. As expected, among the isopeptidases, the proteasome components UCH-L5 and USP14 show higher abundance. Surprisingly, USP14 in certain cell lines is much more abundant than UCH-L5. USP1, USP18 and USP33 are quite rare proteins. USP18, being interferon-inducible was almost undetectable.

**Table 1:** Candidate 2c-biotin targets identified by mass-spectrometry

Database/Gene	Protein	2c-biotin		2c-biot/NAC	
		P.S.	P.N.	P.S	P.N.
TBA1B	Tubulin alpha-1A/1B chain	313	6	95	2
KPYM/PKM	Pyruvate kinase PKM	184	4	nd	nd
BAD96752.1	Beta actin variant	180	5	163	4
RL40	Ubiquitin-60S ribosomal protein L40	140	3	94	1
IF4A1/EIF4A1	Eukaryotic initiation factor 4A-I	128	2	nd	nd
AAF36537.1/EEF1A1	glucocorticoid receptor AF-1 specific elongation factor, partial	113	3	nd	nd
TBB5	Tubulin beta chain	107	2	nd	nd
HS90B	Heat shock protein HSP 90-beta	87	2	nd	nd
COF1	Cofilin-1	83	2	nd	nd
IF5A1/EIF5A1	Eukaryotic translation initiation factor 5A-1	81	1	nd	nd
AAA36787.1	ubiquitin precursor, partial	78	1	nd	nd
PRDX1	Peroxiredoxin-1	76	1	nd	nd
MYL6B	Myosin light chain 6B	66	1	nd	nd
CYB5B	Cytochrome b5 type B	65	1	nd	nd
EAW53693.1/RACK1	guanine nucleotide binding prot., beta polypeptide 2-like 1, isoform CRA_b	65	1	nd	nd
ALDOA	Fructose-bisphosphate aldolase A	61	1	nd	nd
MPCP/SLC25A3	Phosphate carrier protein, mitochondrial	56	1	nd	nd
ENOA/ENO1	Alpha-enolase	55	1	nd	nd
NDKA/NME1	Nucleoside diphosphate kinase A	51	1	nd	nd
ANXA2	Annexin A2	51	1	nd	nd
HSP7C/HSAP8	Heat shock cognate 71 kDa protein	50	1	nd	nd
RL11/RPL11	60S ribosomal protein L11	48	1	nd	nd
G3P/GAPDH	Glyceraldehyde-3-phosphate dehydrogenase	46	1	nd	nd
TPM1	Tropomyosin alpha-1 chain	45	1	nd	nd
RS27A	Ubiquitin-40S ribosomal protein S27a	nd	nd	47	1

P.S. indicates the Mascot protein identification score. Only proteins having one or more peptides with a score higher than 44 (identity or extensive homology, *P* < 0.05) were included in the protein list. P.N. indicates the number of peptides identified and assigned to each single protein. ND (non detectable). Pull-down experiments were performed also in the presence of NAC. Proteins that were isolated in the presence of NAC are indicated in gray and considered as contaminant.

Although there is some variability among cell lines and datasets, overall the newly identified 2c-biotin targets represent high abundant proteins (GAPDH, ENO1, and PKM) frequently ranking within the 5% more abundant proteins. The only exception in the investigated cell lines was myosin light chain 6B MYL6B.

Interestingly, among the 2c targets we identified Cofilin-1. This protein is of particular interest since Cofilin-1 is involved in mediating cytoskeletal changes and cell death in response to a 2c analogous^25^.

### Cofilin-1 phosphorylated at serine 3 interacts with 2c-biotin

**Fig. 5:**
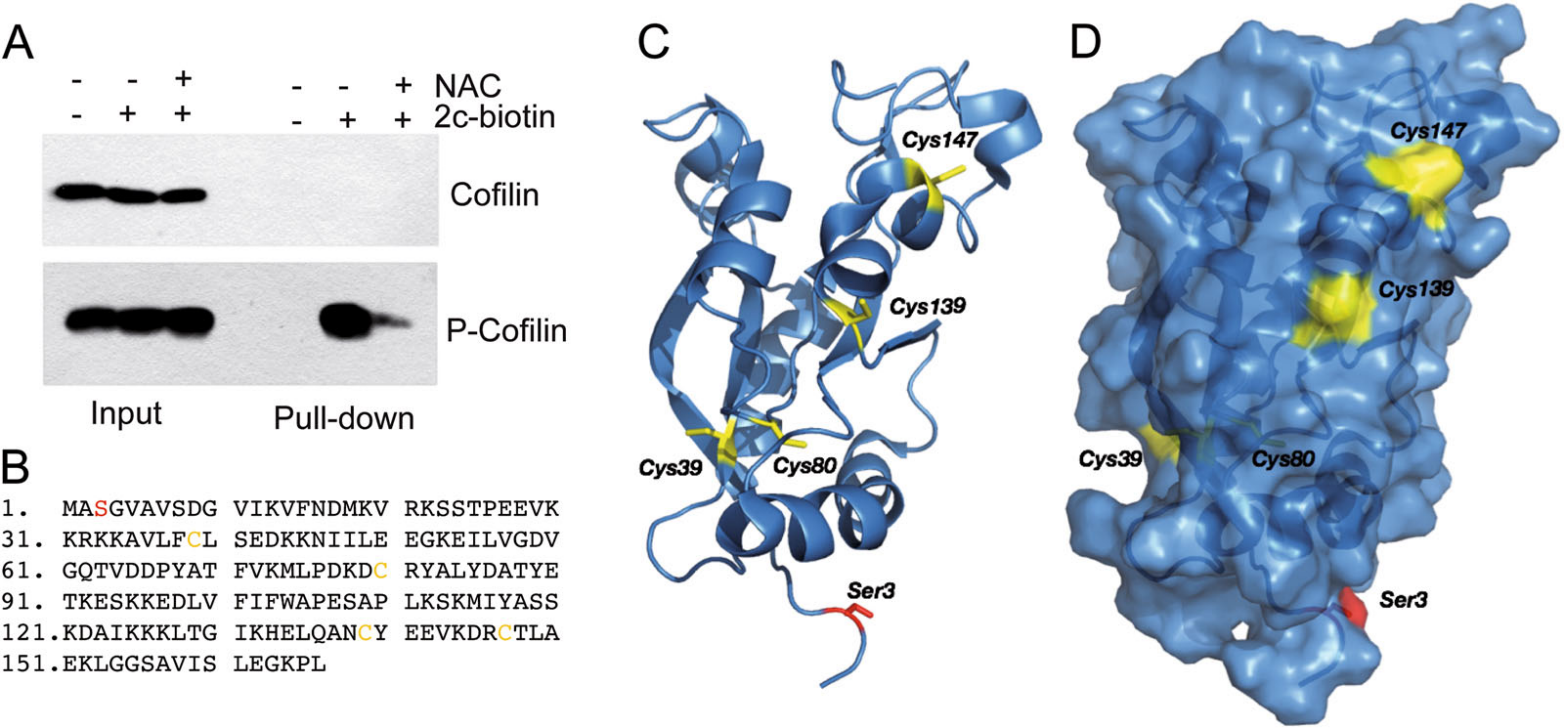
Only the phosphorylated version of Cofilin-1 reacts with 2c. **a** IMR90-E1A cells were treated for 2 h with 10 µM 2c-biotin or left untreated. As a control, 2c-botin was pre-incubated with 5 mM NAC. Cellular proteins were purified by 2c-biotin pull-down, next immunoblotting were performed using the indicated antibodies. Inputs are for enrichment comparison. **b** Primary sequence of human Cofilin-1. Cysteine residues are highlighted in yellow and serine 3 in red. **c** Cartoon structure of Cofilin-1 (PDB: 1Q8G) showing the phosphorylation site (Ser3) in red and the four cysteine residues in yellow. **d** Protein surface superimposed to the cartoon representation, with the same color code. The distance between Ser3 and the available cysteines is 26 Å (Cys139) and 34 Å (Cys147). Images were obtained with the PyMOL Molecular Graphics System, Schrödinger, LLC

To corroborate the proteomic data, we performed 2c-biotin pull-down experiments. We focused the analysis on Cofilin-1, for the above-mentioned reasons. Surprisingly, 2c-biotin pull-down experiments did not evidence any interaction with Cofilin-1 (Fig. 5a). Importantly, Cofilin-1 activities are regulated by phosphorylation of serine 3. Therefore, we performed pull-down experiments using an antibody recognizing Cofilin-1 when phosphorylated at serine 3. Figure 5a indicates that 2c-biotin preferentially reacts with the phosphorylated pool of Cofilin-1.

Cofilin-1 has 4 cysteines at positions 39, 80, 139, and 147 (Fig. 5b). Figure 5d highlights that two cysteine residues (Cys139, Cys147) are located on the surface and are thus potentially available for the reaction with 2c. The other two residues are partially (Cys39) and completely buried (Cys80).

## Discussion

Diaryldienone-derivatives comprise an interesting and heterogeneous family of molecules, which selectively elicit apoptosis in neoplastic cells. Marks of their activities are induction of the UPR, oxidative stress, and the accumulation of poly-ubiquitylated proteins. These multiple cellular responses have been attributed to their ability to function as Michael acceptors against the catalytic cysteine of isopeptidases. Several groups have demonstrated the ability of these molecules to impact on the activity of these enzymes^1–8,10,26^. However, the presence of additional targets has been underestimated.

To clarify this point and to better understand the behavior of these molecules, we generated a biotinylated version of 2c, a diaryldienone-derivative. Our studies demonstrate that addition of biotin does not perturb the activity of 2c. Induction of cell death, activation of caspases, accumulation of poly-ubiquitylated proteins, induction of the anti-redox and of the UPR response were comparable between the original 2c and its biotinylated version. Studies in vivo indicated that the compound is rapidly up-taken by the cells. It shows a diffuse localization and accumulates in the nuclei. Absence of punctate staining points against an uptake through vesicle-mediated trafficking. These data are in agreement with previous hypotheses based on studies with a structurally related compound^18^.

Biotin pull-down experiments demonstrated that 2c covalently binds several isopeptidases, in a NAC-dependent manner and with higher affinity compared to different control proteins. Although these studies need further validation using different essays, we observed a higher binding affinity for USP1 and USP33 compared to USP14, USP18, or UCH-L5. We cannot exclude that these differences stem from a different in vivo accessibility of the enzymes, due to the existence of multi-protein complexes.

Surprisingly, the serine/threonine kinase AKT was bound with high affinity by 2c-biotin. AKT is an important signaling enzyme, regulating survival and proliferation, whose activity is affected by G5, a 2c-like compound^25^. AKT kinase activity depends on two critical residues; threonine 308 and serine 437. Treatment with G5 promotes a transient phosphorylation of threonine 308, followed by its dephosphorylation. By contrast, serine 473 is dephosphorylated at earlier time-points. While dephosphorylation of threonine 308 is PP2A-dependent, serine 473 dephosphorylation is largely independent^25^. Interestingly, cysteine residues 310 and 296 found in the T-loop region are critical for AKT activity and can be subjects of bioreductive alkylation by pyranonaphthoquinone lactones^27,28^. Hence, AKT contains 2c-addictable cysteines critical for its activity. Further study will be necessary to understand whether the reaction with 2c or similar compounds impairs AKT enzymatic activity.

The mass-spec analysis confirmed that additional cellular proteins are modified by 2c-biotin. These proteins can be grouped in five major functional classes (metabolism, proteostasis, cytoskeleton, signaling, and oxidative stress).

Previous studies indicated that these compounds can be relatively promiscuous. Actin can be addicted by 15-Deoxy-Δ^12,13^-prostaglandin J_2_ that contains the same unsaturated ketone pharmacophore of 2c^29^. The bis(benzylidene)piperidone RA190 reacts with cysteine 88 of the proteasome ubiquitin receptor hRpn13 and MCB-613 (4-ethyl-2,6-bis-pyridin-3-ylmethylene-cyclohexanone), a molecule structurally similar to 2c, can stimulate steroid receptor co-activators (SRCs) transcriptional activity^7,30^.

Surprisingly, isopeptidases were not identified by this analysis. It should be noted that, among the identified 2c targets, high abundance proteins are the foremost represented. In principle, these high abundance targets could compete with high affinity but low abundant targets, such as USP1 and USP33.

2c and similar molecules trigger profound changes of cytoskeleton and cell morphology. Time-lapse experiments demonstrated that, before the appearance of apoptosis, stress fibers disassemble and cells lose adhesion^31^. An important contribution to these cytoskeletal changes is provided by Cofilin-1. Cofilin-1 dephosphorylation triggers F-actin disassembling and 2c can favor this dephosphorylation^25^. Importantly, 2c-biotin preferentially modifies the phosphorylated pool of Cofilin-1. This observation suggests that 2c could impact F-actin dynamic directly, by hijacking Cofilin-1 functions. Cofilin dimers/oligomers exhibit bundling activities and dimerization occurs via the formation of intermolecular disulfide bridges involving Cys (39) and, probably, Cys (147) of adjacent Cofilin units. Indeed, these cysteines can react with cucurbitacins, triterpenoid natural compounds^32^. Smilarly, by reacting with the cysteines of Cofilin, 2c might impair dimerization and thus impact directly on F-actin severing^33–35^. Cysteine residues (Cys139, Cys147) are located on the surface and are thus potentially available for the reaction with 2c. Interestingly, the distance between Ser3 and these cysteines is 26 Å (Cys139) and 34 Å (Cys147).

In summary, our studies demonstrate that diaryldienone-derivatives can be addicted to several cellular proteins in addition to isopeptidases. The combination of different targets can elicit multiple stresses, which are not manageable in neoplastic cells, thus explaining their efficient anti-tumor activity observed in vivo.

## Materials and methods

### Reagents and antibodies

Chemicals used were: bortezomib (LC Laboratories), Propidium Iodide and N-acetylcysteine (Sigma Aldrich). Primary antibodies used were: anti-Gas2^36^ anti-actin (Sigma/Aldrich) anti-USP1^37^ anti-USP33 (Millipore) anti-AKT (Cell Signaling) anti-UCH-L5 (Abcam), anti-USP18^38^ anti-HDAC4^39^ anti-MEF2D (BD Bioscience), anti-GRP78, anti-USP14, anti-Cofilin-1, anti-pCofilin-1 (Santa Cruz Biotechnologies).

### Synthesis of the biotinylated version of 2c

Briefly, the biotinylated derivative of 2c was obtained by the insertion of a cadaverine linker between biotin and 2c. To this end, biotin was first used to acylate one of the amino groups of cadaverine, and then the resulting adduct was linked to 2c via a carbamate linkage obtained by reaction with 2c-OSu. Products and intermediates were characterized by Electrospray Ionization mass spectrometry (ESI-MS) performed on an Esquire 4000 (Bruker Daltonics) spectrometer and by ^1^H and ^13^C NMR (Varian, 500 MHz). Full details are reported in the supplementary materials.

### Culture conditions, fluorescence microscopy, and cell death analysis

MEC-1 cells were grown in RPMI medium whereas IMR90-E1A cells were cultured in DMEM. Mediums were supplemented with heated inactivated fetal calf serum (Sigma Aldrich), penicillin (100 U/mL), glutamine (2 mmol/L), and streptomycin (100 μg/mL). Fluorescence microscopy was performed as previously described^1^. Briefly, cells were fixed in 3% paraformaldehyde, permeabilized with 1% Triton-X100 and incubated with TRITC streptavidin (Molecular probes). For the quantification of cell death, cells were re-suspended in 500 μL of PBS and stained with propidium iodide (PI). Fluorescence was determined with a FACScan™ (Beckman Dickinson). IFN-α2a (1000 units/mL; Jena Bioscience) was used as previously described^40^.

### 2c-Biotin pull-down and immunoblotting

For pull-down experiments 4.5 × 10^5^ IMR90-E1A cells were treated with 10 µM 2c-biotin for 2 h. When used, N-acetylcysteine (NAC) was 5 mM. Cell lysis was performed in lysis buffer (PBS, 2%SDS, 5 mM DTT). Proteins obtained after an SDS denaturating lysis and sonication, were incubated with streptavidin-agarose beads (GE Healthcare). After several washes in lysis buffer, they were eluted after boiling in sample buffer. Next SDS/PAGE electrophoresis was performed and proteins were transferred to nitrocellulose membrane and incubated with the specific primary antibodies. Secondary antibodies were peroxidase-conjugated goat anti-rabbit or anti-mouse (Sigma/Aldrich). Blots were developed with Super Signal West Dura (Pierce).

### RNA extraction and qRT-PCR analysis

RNA was extracted using Tri-Reagent (Molecular Research Center) and retrotranscribed by using 100U of Moloney murine leukemia virus reverse transcriptase (Invitrogen). The primer sequences used are available upon request. Quantitative reverse transcription-PCR (qRT-PCR) analyses were performed using Bio-Rad CFX96 and SYBR Green technology. HPRT and GAPDH were used as normalizer genes. All reactions were done in triplicate. Data were from al least three experiments ±SD.

### Proteomic analysis

2c–biotin pull down samples were separated by SDS—PAGE and the gel stained by Coomassie blue. The lanes corresponding to 2c—biotin and 2c—biotin plus NAC were excised and divided into five parts. Proteins were in gel digested and identified by LC-MS/MS analysis essentially as previously described using a 1200 Series Nano HPLC (Agilent)^41^. Only proteins having one or more peptides with a score higher than 44 (identity or extensive homology, *P* < 0.05) were included in the protein list. Keratins and Streptavidin were excluded from the identified proteins. Protein have been listed according to the Mascot protein score^42,43^.

## Electronic supplementary material

Supplementary Information
