# Supplementary material for: The binding landscape of a partially-selective isopeptidase inhibitor with potent pro-death activity, based on the bis(arylidene)cyclohexanone scaffold
Source: Cell Death Dis. 2018 Feb 7;9(2):184. doi: 10.1038/s41419-017-0259-1 (PMC5833369; doi:10.1038/s41419-017-0259-1)
Supplement: Supplementary file 1 — Supplementary Information [file 41419_2017_259_MOESM1_ESM.docx]

**Supplementary Material**

**General:** Thin layer chromatography (TLC) were conducted on Merck pre-coated plastic sheets with 0.25mm silica gel 60F-254. TLC plates were stained with aqueous permanganate solution, or iodine vapors, or examined under ultraviolet light UV (λ=254 and λ=365). Melting points **(mp)** were measured on a Büchi SMP-20 apparatus. Nuclear magnetic resonance **(NMR)** 500 MHz ^1^H-NMR and 125.4 MHz ^13^C-NMR spectra were obtained on a Varian 500 spectrometer. 400 MHz ^1^H-NMR and 100 MHz ^13^C-NMR spectra were obtained on a Jeol JNM-EX400 instrument. 270 MHz ^1^H-NMR and 67.5 MHz ^13^C-NMR spectra were obtained on a Jeol JNM-EX270 spectrometer. Chemical shifts are reported in ppm using the solvent residual signal as an internal reference (ex. CDCl_3_: δH = 7.26 ppm, δC = 77.16 ppm). Coupling constants (J) are given in Hz. Electrospray Ionization **(ESI)** mass spectrometry measurements **(MS)** were performed on a Esquire 4000 (Bruker Daltonics) spectrometer.

Chemicals were purchased from Sigma-Aldrich-Fluka and used as received. Solvents were purchased from Sigma-Aldrich-Fluka, and deuterated solvents from Sigma-Aldrich-Fluka and Cambridge Isotope Laboratories. Dichloromethane was dried over CaCl_2_; pyridine, Acetonitrile were bought as anhydrous from Sigma Aldrich. Other solvents were of synthetic grade. Reaction flasks were flame dried and placed under vacuum using a Schlenk line and purged with Ar. To adjust the reaction flask’s necks, silicon stoppers were used, and continuous flux of Argon was used to keep the inert atmosphere.

***tert-*butyl N-(5-aminopentyl)carbamate (1).** A solution of di-*tert*-butyl dicarbonate (3.6 g, 16 mmol) in dichloromethane (30 mL) was added dropwise to a solution of 1,5-diaminopenthane (9 g, 88 mmol) in dichloromethane (60 mL) at 0°C. The solution was stirred for 1 hour at 0°C and for another hour at room temperature. The reaction mixture was filtered off to remove a white precipitate and the solvent was removed under reduced pressure. The residue was dissolved in ethyl acetate (20 mL) and washed with a saturated solution of sodium hydrogen carbonate and brine. The organic phase was dried over anhydrous sodium sulfate and then the solvent was removed to give a yellow oil **(1)**, 1.75 g ; 56%; **^1^H NMR** (DMSO-d_6_): δ 1.35-1.51 (*m*, 6H), 1.44 (*s*, 9H) 2.67-2.70 (*m*, 2H, J_1_=7.1 Hz, J_2_=3.1 Hz), 3.13 (*m*, 2H), 4.56 (*s*, 1H); **^13^C-NMR** (DMSO-d_6_): δ 23.83, 28.43, 29.79, 30.29, 40.26, 41.56, 77.54, 156.17; **ESI-MS** (m/z): 203 [MH^+^].

***tert*-butyl-5-(5-((3aR,6S,6aS)-hexahydro-2-oxo-1H-thieno-[3,4-d]-imidazol-6-yl)pentanamido) pentylcarbamate (2).** Biotin (1 g, 4.09 mmol) was dissolved under anhydrous atmosphere in 30 mL of dry acetonitrile, and the pH of the solution was adjusted to 8.5 with triethylamine. The solution was cooled at 0°C, 1.14 g of EDC-Cl (7.36 mmol) and 0.83 g of HOBt 6.13 mmol) were added, then the mixture was stirred for 30 minutes. After this time the pH was newly adjusted to 8.5 by adding triethylamine, and 1.16 g of **(1)** were added (5.73 mmol). The mixture was stirred for 1 hour at 0°C and then for 16 hours at room temperature. After this time the mixture was filtered off, the solvent was removed, the solid residue was divided between ethyl acetate and distilled water and the organic phase was washed with 10% citric acid, saturated solution of sodium hydrogen carbonate and brine. The organic layer was dried over anhydrous sodium sulfate and the solvent was removed by distillation under reduced pressure in order to give a brown product **(2)**, 0.79 g; 45%; **^1^H NMR** (DMSO-d_6_ δ 1.18-1.65 (*m*, 21H), 2.18 (*t*, 2H, J=7.2 Hz), 2.69 (*m*, 1H,), 2.91 (*m*, 1H,), 3.02 (*t*, 2H, J=6.4 Hz), 3.17 (*m*, 3H), 4.34 (*m*, 1H), 4.53 (*m*, 1H), 6.36 (*s*, 1H), 6.42 (*s*, 1H), 7.73 (*s*, 1H), 7.78 (*m*, 1H). **^13^C-NMR** (DMSO-d_6_): δ 23.45, 24.40, 24.47, 25.90, 28.60, 29.41- 29.44, 31.91, 40.4, 42.9, 55.99, 59.75, 60.51, 61.60, 79.5, 156.0, 164.7, 172.7; **ESI-MS** (m/z): 451.1 [MNa^+^].

**N-(5-aminopentyl)-5-((3aR,6S,6aS)-hexahydro-2-oxo-1H-thieno-[3,4-d]-imidazol-6-yl) pentanamide (3).** Compound **(2)** was dissolved in the minimum amount of a 1:1 mixture of trifluoroacetic acid/dichloromethane, and the solution was stirred for 2 hours at room temperature. After this tim,e 20 mL of dichloromethane were added and all the solvents were removed under vacuum in order to obtain a brown solid **(3)**, 0.62 g; 100%; **^1^H NMR** (DMSO-d_6_): δ 1.16-1.60 (*m*, 12H), 1.97-2.07 (*m*, 2H), 2.55-2.58 (*m*, 2H), 2.75-2.84 (*m*, 2H), 2.97-3.08 (*m*,3H), 4.01-4.30 (*m*, 2H), 6.42 (*bs*, 2H), 7.70 (*bs*, 1H); **^13^C-NMR** (DMSO-d_6_): δ 24.07, 25.24, 25.51 , 25.91, 29.06, 34.04, 39.63, 42.77, 53.34, 56.23, 58.53, 70.20, 173.66, 174.98; **ESI-MS** (m/z): 329 [MH^+^].

**(3E,5E)-3,5-bis-(4-nitro-benzyliden)-4-oxocyclohexyl-2,5-dioxopyrrolinidyl-1-carbonate (2c-Osu)** N,N’-disuccinimidyl carbonate (0.61g, 2.38 mmol) was added to a solution of **(2c)** (0.45 g, 1.18 mmol) in a 1:1 mixture of anhydrous dichloromethane/acetonitrile; anhydrous pyridine was added until the solution become clear. After stirring under an argon atmosphere for 18 hours at room temperature, the product was precipitated by ethyl ether in an ice-bath, filtered and dried to give a yellow solid **(2c-Osu)**. 0.47 g; 76%**; MP**: 172-175 °C**; ^1^H NMR** (DMSO-d_6_): δ 2.71 (*s*, 4H), 3.33 (*m*, 4H), 5.34 (*m*, 1H), 7.78-8.28 (*d*, 4H), 7.87 (*s*, 2H); **^13^C-NMR** (DMSO-d_6_): δ 25.26, 32.5, 75, 123.62, 131.21, 133.86, 136.74, 141.26, 147.13, 151, 169.61, 187.

**(3E,5E)-3,5,bis(4-nitrobenzylidene)-4-oxocyclohexyl 5-(5-((3aR,6S,6aS)-hexahydro-2-oxo-1H-thieno[3,4-d]imidazol-6-yl)pentanamido)pentylcarbamate (Biotinyl-2c).** 0.15 g of compound **(3)** were dissolved in a mixture of dicholormethane (8 mL) and pyridine (3 mL), and the pH of the solution was adjusted to 8.5 with triethylamine. When compound **(3)** was completely dissolved, 0.141 g of compound **(2c-Osu)** were added. After stirring at room temperature for 18 hours, the product was precipitated by adding ethyl ether in ice-bath, filtered and dried to give a brown solid **(Biotinyl-2c)**, 0.113 g ; 57%**, ^1^H NMR** (DMSO-d_6_): δ 1.05-1.60 (*m*, 12H), 1.97-2.05 (*m*, 2H), 2.77-2.88 (*m*, 2H), 3.33 (*m*, 4H), 4.05-4.30 (*m*, 2H), 5.00 (*m*, 1H), 6.33-6.38 (*d*, 2H), 6.98-7.10 (*m*, 2H), 7.78-8.28 (*d*, 4H). **^13^C-NMR** (DMSO-d_6_): δ 23.48, 25.19, 25.28, 26.59, 28.1, 28.78, 32.48, 35.18, 45.57, 52.04, 55.46, 59.23, 62.61, 66.47, 123.81,131.43, 135.68, 135.92,141.74, 147.25, 155.42, 162.94, 172.03, 187.59; **ESI-MS** (m/z): 735.2 [MH^+^], 757.2 [MNa^+^]
